# Identification and verification of PTPN3 as a novel biomarker in predicting cancer prognosis, immunity, and immunotherapeutic efficacy

**DOI:** 10.1186/s40001-023-01587-5

**Published:** 2024-01-03

**Authors:** Ziting Zhou, Zhengjun Lin, Mingrui Wang, Lifan Wang, Yuqiao Ji, Jing Yang, Yaocheng Yang, Guanghui Zhu, Tang Liu

**Affiliations:** 1 Department of Orthopedics, The Second Xiangya Hospital of Central South University, Changsha, 410011 Hunan China; 2 Department of Pediatric Orthopedics, Hunan Provincial Key Laboratory of Pediatric Orthopedics, Hunan Children’s Hospital, Changsha, 410007 Hunan China; 3 Furong Laboratory, Changsha, Hunan China; 4 MOE Key Lab of Rare Pediatric Diseases, University of South China, Hengyang, 421001 Hunan China; 5 School of Basic Medicine Science, Central South University, Changsha, 410078 Hunan China

## Abstract

**Background:**

The importance of protein tyrosine phosphatase non-receptor type 3 (PTPN3) in controlling multifaceted tumor cell behaviors throughout cancer development has received widespread attention. Nevertheless, little is known about the biological roles of PTPN3 in drug sensitivity, immunotherapeutic effectiveness, tumor immune microenvironment, and cancer prognosis.

**Methods:**

The Cancer Genome Atlas (TCGA) database's RNAseq data were used to examine the expression of PTPN3 in 33 different cancer types. In addition, immunohistochemistry (IHC) was performed to validate the expression of PTPN3 across various cancer types within our clinical cohorts. The features of PTPN3 alterations were demonstrated throughout the cBioPortal database. This study focused on examining the prognostic and clinicopathological importance of PTPN3 through the acquisition of clinical data from the TCGA database. The investigation of PTPN3's probable role in the tumor immune microenvironment was demonstrated by the application of CIBERSORT, ESTIMATE algorithms, and the TISIDB database. Using Spearman's rank correlation coefficient, the relationships between PTPN3 expression and tumor mutation burden (TMB) and microsatellite instability (MSI) were evaluated. To further investigate the putative biological activities and downstream pathways of PTPN3 in various cancers in humans, Gene Set Enrichment Analysis (GSEA) was carried out. In addition, an examination was conducted to explore the associations between PTPN3 and the effectiveness of PD-1/PD-L1 inhibitors, utilizing data extracted from the GEO database.

**Results:**

PTPN3 was abnormally expressed in multiple cancer types and was also strictly associated with the prognosis of cancer patients. IHC was used to investigate and confirm the various expression levels of PTPN3 in various malignancies, including breast cancer, lung cancer, sarcoma, and kidney renal clear cell carcinoma in our clinical cohorts. There is a high correlation between the levels of PTPN3 expression in different cancers and infiltrating immune cells, including mast cells, B cells, regulatory T cells, CD8 + T cells, macrophages, and dendritic cells. Infiltrating immune cells, such as regulatory T cells, CD8 + T cells, macrophages, B cells, dendritic cells, and mast cells, are strongly correlated with PTPN3 expression levels in various tumors. The expression of PTPN3 exhibited a substantial correlation with many immune-related biomolecules and the expression of TMB and MSI in multiple types of cancer. In addition, PTPN3 has demonstrated promise in predicting the therapeutic benefits of PD-1/PD-L1 inhibitors and the susceptibility to anti-cancer medications in the treatment of clinical cancer.

**Conclusions:**

Our findings highlight the importance of PTPN3 as a prognostic biomarker and predictor of immunotherapy success in various forms of cancer. Furthermore, PTPN3 appears to have an important role in modifying the tumor immune microenvironment, highlighting its potential as a promising biomarker for prognosis prediction, immunotherapeutic efficacy evaluation, and identification of immune-related characteristics in diverse cancer types.

## Introduction

Cancer is the leading cause of death and a worldwide barrier to quality of life. Although various immunotherapeutic methods have achieved long-term clinical responses, their efficacy varies between patients, and they are only effective in certain subsets of cancer patients [1]. One of the most important reasons is that tumors range greatly in terms of the origin and genetic alterations. Despite the differences, pan-cancer research can shed light on shared pathogenesis among cancers, such as key driver mutations, altered signaling pathways, and immune infiltration in the tumor microenvironment (TME), implying the intriguing possibility of targeting common traits among various cancers with similar therapeutic approaches [2]. Therefore, identifying novel biomarkers associated with cancer immunology that can predict the potential for immunotherapy efficacy and the best immunotherapy combinations for the individual patient are high priorities for clinical development.

PTPN3, also known as PTPH1, is a member of the non-transmembrane protein phosphatase (PTP) family [3], which is known to be involved in diverse physiological and pathological processes, such as hematopoiesis, inflammation, cell differentiation, and oncogenic transformation [4–7]. It contains an N-terminal FERM domain followed by a single PDZ domain and the C-terminal PTP domain [8]. Currently, several studies have demonstrated that PTPN3 is expressed differentially in malignancies and is crucial for cancer progression and prognosis. Multiple lines of evidence have indicated that PTPN3 may function as a potential tumor suppressor gene in several cancers. PTPN3 was initially regarded as a potential tumor suppressor in colorectal cancer (CRC) [9]. In lung cancer, PTPN3 has been shown to limit cellular proliferation and invasion by increasing EGFR endocytic degradation [10, 11]. PTPN3 is favorably correlated with a better prognosis in perihilar cholangiocarcinoma and to slow down the growth of the malignancy by blocking AKT phosphorylation [12, 13]. However, PTPN3 may have a dual role in the development of various malignancies. Several investigations provided strong evidence that PTPN3 plays an oncogenic role in a variety of malignancies [14]. Evidence has indicated that PTPN3 can enhance metastasis and invasion in several cancer types, such as breast cancer, ovarian cancer, and gastric cancer [15]. Regarding gastric adenocarcinoma, there was a considerable upregulation of PTPN3, which was linked with the pathological grade [16]. In ovarian cancer cells resistant to cisplatin and doxorubicin, PTPN3 is significantly elevated and can support chemotherapy resistance as well as cancer stemness [17]. Moreover, several studies have reported that PTPN3 can function as prognostic biomarkers to predict chemotherapeutic responses [18–20]. Overall, PTPN3 has a variety of effects on the progression and prognosis of cancer, but the underlying processes are not well-understood. Comprehensive pan-cancer studies that explicitly target PTPN3 have not yet been conducted, and the precise roles that PTPN3 plays in modifying the tumor immune milieu and how that affects immunotherapy have not been thoroughly examined in scientific literature. A comprehensive pan-cancer study may contribute to elucidating the functions of PTPN3 in immunity, immunotherapy efficacy, and cancer prognosis prediction, opening up new avenues for the discovery of novel therapeutic targets.

In the present pan-cancer research, we systematically investigated and verified the PTPN3 expression pattern and its prognostic value. We also looked into the connections between PTPN3, immune cell infiltration, immunological-related biomarkers, and the effectiveness of immunotherapy. This highlighted the possible function of PTPN3 in mediating the tumor immune microenvironment and immunotherapy effectiveness.

## Materials and methods

### Data processing and expression analysis of PTPN3

The RNA expression, somatic mutation, and clinical data of The Cancer Genome Atlas (TCGA) were obtained by accessing the UCSC Xena database (https://xenabrowser.net/datapages/). We used the Strawberry Perl (Version 5.32.1.1, http://strawberryperl.com/) tool to obtain PTPN3 gene expression data. Data analysis was conducted using R software (Version 4.0.2). The differential expression of PTPN3 between tumor and normal samples was assessed using a Student *t* test, and the results were visualized using the R package "ggplot2".

### Analysis of PTPN3 genetic alterations

The cBioPortal database (http://www.cbioportal.org/) was utilized for the analysis of genetic alterations of PTPN3 within the TCGA pan-cancer data sets. Alteration frequency, mutation type, and mutated site information were obtained and applied to explore the mutation pattern of PTPN3 in pan-cancer.

### Prognostic value of PTPN3

The correlations of PTPN3 with overall survival (OS), progression-free survival (PFS), disease-free survival (DFS), and disease-specific survival (DSS) were thoroughly evaluated using univariate Cox regression models. Kaplan–Meier analysis was performed to evaluate the different survival time between patients with high or low PTPN3 expression. The R-packages "survival", "survminer", "limma", and "ggpubr" were employed to perform the analysis. The log-rank *p* value and hazard ratio (HR) with 95% confidence intervals were calculated. All the conducted analyses were deemed statistically significant at *p* < 0.05.

### Gene set enrichment analysis (GSEA)

To examine the biological functions and signaling pathways involving PTPN3 in pan-cancer, gene set enrichment analysis (GSEA) was carried out. We performed GSEA to discover potential pathways by Kyoto Encyclopedia of Genes and Genomes (KEGG) and gene ontology (GO) terms affected by PTPN3 using R packages “limma”, “org.Hs.eg.db”, “enrichplot” and “clusterProfiler”.

### Relationship between PTPN3 expression and immune landscape

The Estimation of Stromal and Immune cells in Malignant Tumors using the Expression Data (ESTIMATE) algorithm was performed to calculate the stroma and immune scores according to PTPN3 expression via the R package “ESTIMATE”. Then, we used CIBERSORT, a metagene tool that can quantify the putative proportion of immune cell fraction from gene expression profiles, to illustrate the immune cell infiltration in different cancers. The investigation of correlations between PTPN3 and the expression of immune-related biomolecules in pan-cancer was conducted by the TISIDB database (http://cis.hku.hk/TISIDB/index.php).

### Correlation of PTPN3 expression with tumor mutation burden (TMB), tumor microsatellite instability (TMI)

The correlations of clinical prognosis and related genes, of which TMB and MSI scores.TMB, an immune-response biomarker that quantifies the immunotherapeutic response of PD-1 antibodies. MSI is caused by MMR deficiency and is related to the clinical outcomes of patients. Results were analyzed by Spearman‘s rank correlation coefficient and then presented as Radar plots by R-package “fmsb”.

### Analysis of PTPN3 expression association with the immunotherapy efficacy

To investigate the correlation between PTPN3 expression levels and the effectiveness of immunotherapy, three databases, including GSE67501, GSE78220, and Imvigor, were obtained from the Gene Expression Omnibus (GEO), accessible at https://www.ncbi.nlm.nih.gov/geo/. The plots were drawn using R-package “ggpubr” and “ggplot2”.

### Drug sensitivity analysis of PTPN3 in pan-cancer

The NCI-60 compound activity data and RNA-seq expression profiles were obtained from the CellMiner™ online database (https://discover.nci.nih.gov/cellminer/home.do) to investigate the relationship between PTPN3 expression and drug sensitivity across various types of cancer. The “impute”, “ggplot2”, and “ggpubr” R packages were utilized to illustrate associations between PTPN3 and the sensitivity of selected drugs approved by the FDA or clinical trials.

### IHC validation

All human sample slides were baked at 65 °C for 2 h and then were dewaxed and rehydrated. Slides were immersed in EDTA antigen repair buffer, and antigen repair was conducted at 100 °C for 20 min. Endogenous peroxidase was eliminated through incubation with 3% peroxidase solution for 25 min, followed by 5% bovine serum albumin incubation for 20 min at room temperature. Incubation with primary antibodies (PTPN3, Affinity, DF15463, China) was conducted overnight at 4 °C. Then, the samples were incubated with a secondary antibody for 50 min at room temperature. The immunohistochemistry scores were by two individuals blinded to the clinical data.

### Statistical analysis

Differences between groups were analyzed using a Student’s *t* test. The Kaplan–Meier curve, log-rank test, and Cox analysis were used for all survival analyses in this study. Statistical analyses were performed using R 4.1.2. (https://www.Rproject.org). *P* < 0.05 (two-tailed) was considered statistically significant.

## Results

### PTPN3 expression analysis in pan-cancer

**Fig. 1:**
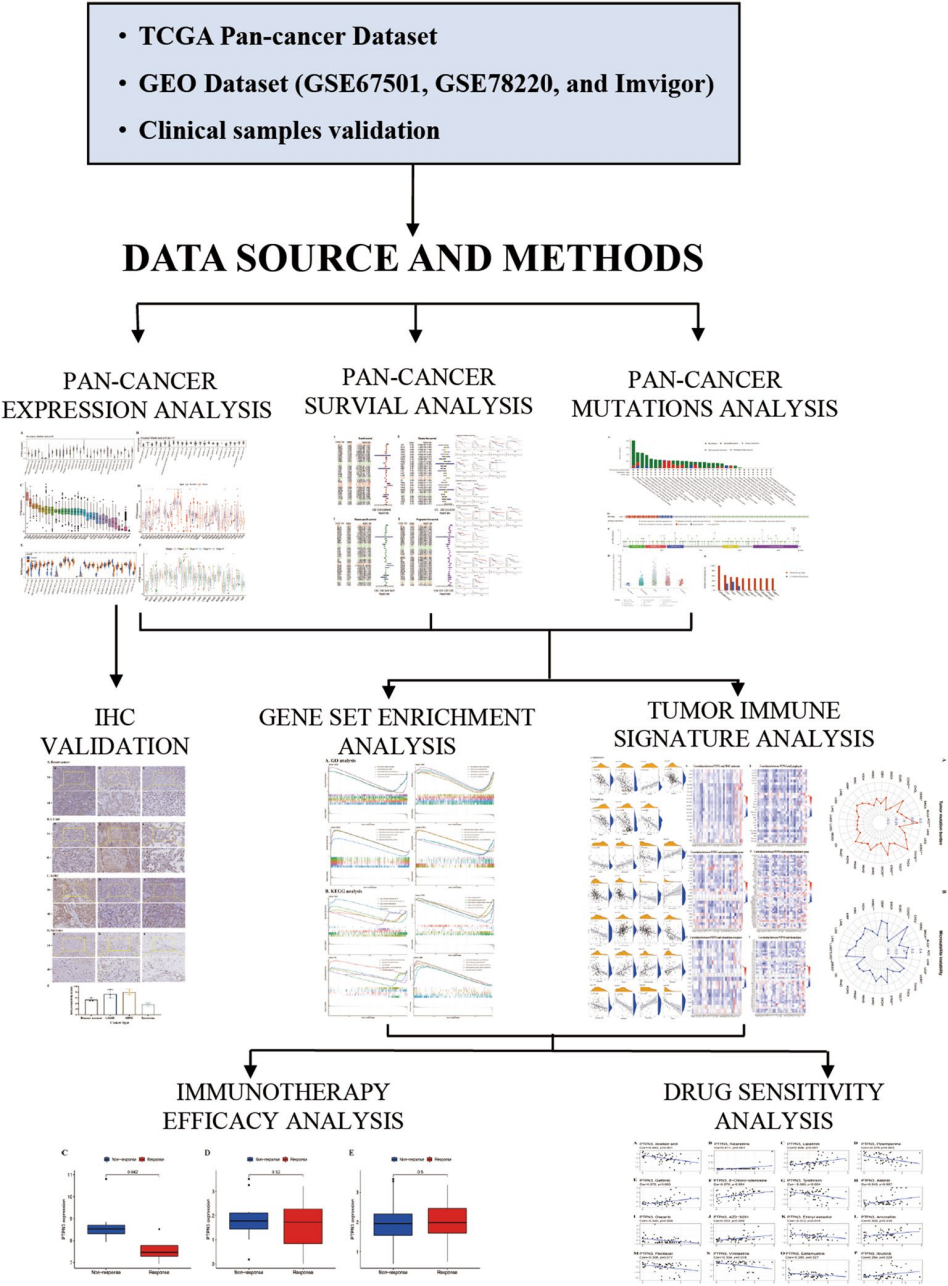
Study flow diagram

**Fig. 2:**
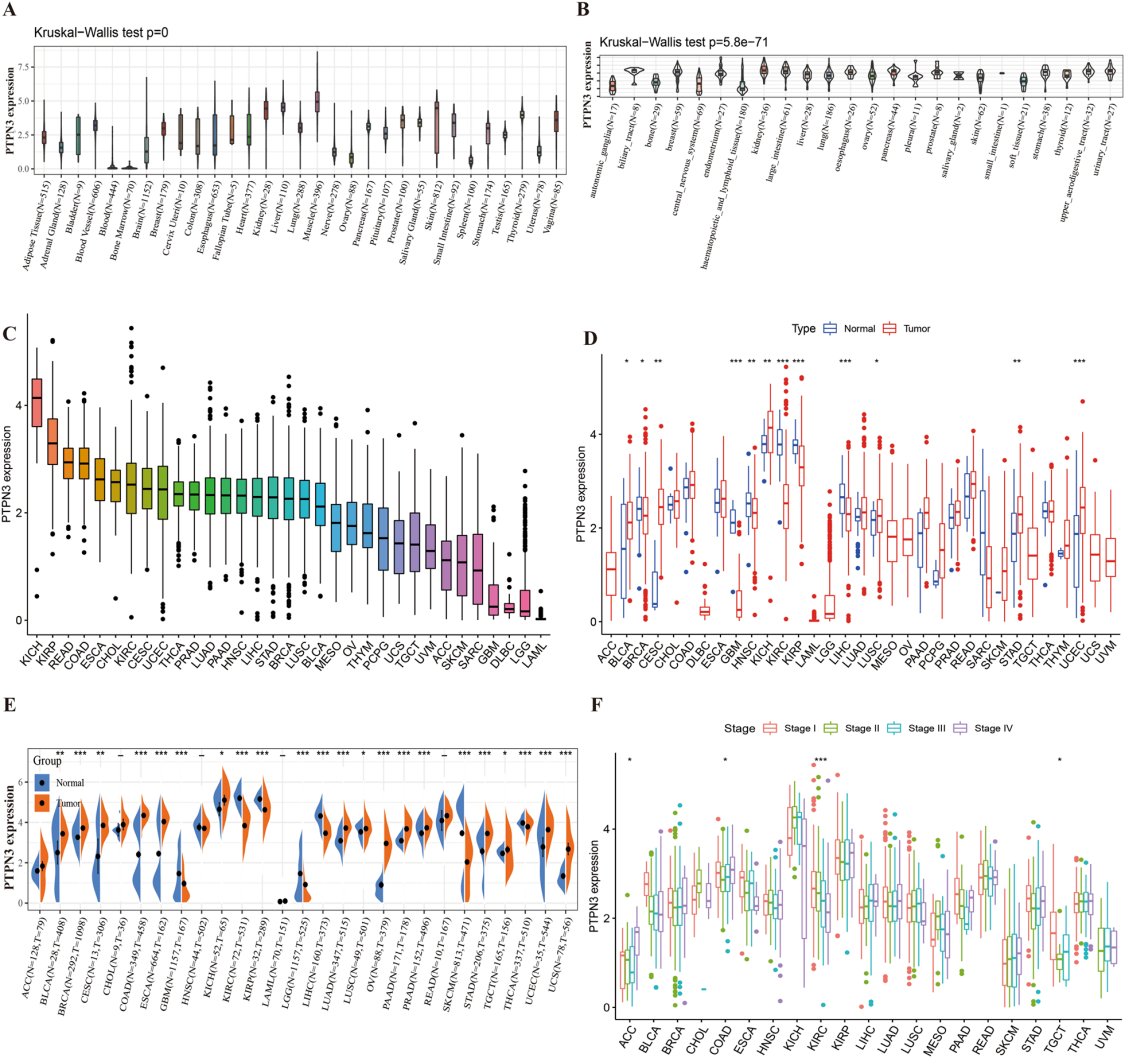
Expression pattern of PTPN3. **A** Expression level of PTPN3 in 31 normal tissues from the GTEx database. **B** Expression level of PTPN3 in 24 tumor cell lines from the CCLE database. **C** Expression level of PTPN3 in pan-cancer. **D** Comparison of PTPN3 expression level between cancer and normal samples from TCGA database. **E** Comparison of PTPN3 expression level between cancer and normal samples from GTEx database. **F** Expression level of PTPN3 in patients with different WHO stages in various cancer from the TCGA database

The study flow diagram is shown in Fig. 1. To investigate the PTPN3 expression in normal human tissues, we analyzed the PTPN3 expression in physiological tissue presented in the GTEx database. The mRNA expression of PTPN3 expression varied in different tissues, and the expression levels of PTPN3 were highest in muscle, liver, and kidney, while PTPN3 was expressed lowly in bone marrow, blood, and spleen compared with other normal tissues (Fig. 2A). We also analyzed the expression of PTPN3 in cancer cell lines provided by the CCLE database (Fig. 2B). We found a high PTPN3 expression in the kidney, pancreas, and urinary tract cancer cell lines. Then, we explored the expression of the PTPN3 in diverse cancers in the TCGA pan-cancer database. We found that PTPN3 expression in KICH was the highest, followed by KIRP, READ, and COAD, while PTPN3 was lowly expressed in DLBC, LGG, and LAML (Fig. 2C). Next, we analyze the differential expression of PTPN3 between tumor and normal tissues in the TCGA pan-cancer data set. The results showed a significant difference in 12 cancers among 24 cancers, excluding those without normal samples. Compared to the corresponding adjacent normal tissues, we found a higher PTPN3 expression in BLCA, CESC, KICH, LUSC, STAD, and UCEC, and a lower PTPN3 expression in BRCA, GBM, HNSC, KIRC, KIRP and LIHC (*p* < 0.05) (Fig. 2D). In addition, we conducted a comparative analysis of the differential expression of PTPN3 in cancer samples and normal samples obtained from the GTEx database. It was shown that the expression of PTPN3 was considerably elevated in cancer samples of BLCA, BRCA, CESC, COAD, ESCA, KICH, LUAD, LUSC, OV, PAAD, PRAD, STAD, TGCT, UCEC, and UCS. Conversely, PTPN3 was found to be significantly downregulated in GBM, KIRC, KIRP, LGG, LIHC, SKCM, and THCA (*p* < 0.05) (Fig. 2E). In addition, we analyzed the stage-specific expressional changes of PTPN3 in pan-cancer. Our results showed that PTPN3 was higher in later clinical stages in ACC and COAD. In contrast, higher PTPN3 expression in earlier stages was observed in KIRC and TGCT (Fig. 2F).

### Genetic alteration analysis of PTPN3 in pan-cancer

**Fig. 3:**
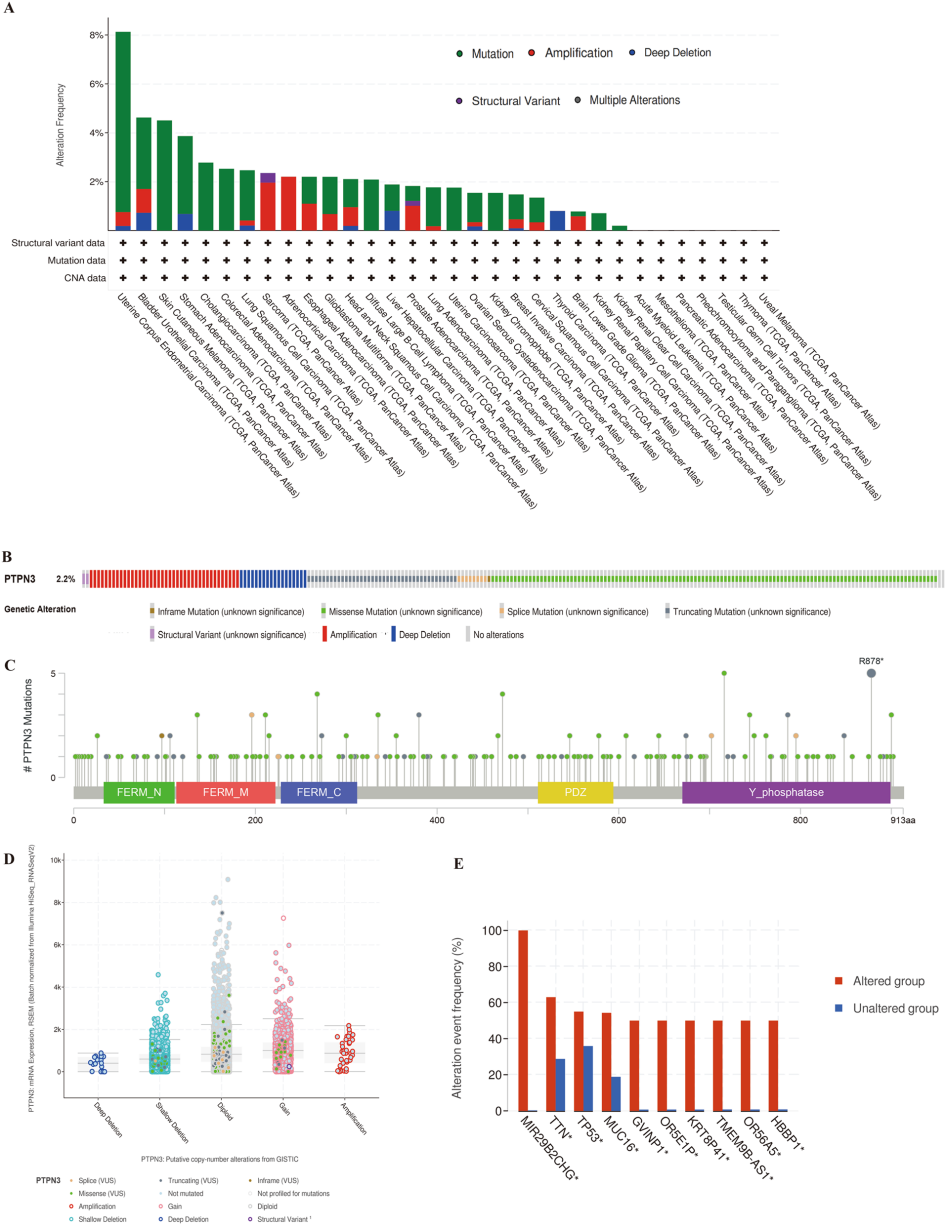
Genetic alteration characteristics of PTPN3 in pan-cancer. **A** Alteration frequency of PTPN3 with different types of mutations in different cancer types. **B** Different genetic alteration types of PTPN3. **C** Mutation types, sites, and sample numbers of the PTPN3 genetic alterations. **D** Correlated alteration types and putative copy-number of PTPN3 in pan-cancer. **E** Co-occurrence of genetic mutations in tumors with PTPN3 alterations

We further investigated the genetic alternation features of PTPN3 in pan-cancer by enrolling in the TCGA Pan-Cancer Atlas studies. The high gene alteration rate of PTPN3 was detected in uterine corpus endometrial carcinoma, bladder urothelial carcinoma, skin cutaneous melanoma, stomach adenocarcinoma, cholangiocarcinoma, colorectal adenocarcinoma, and lung squamous cell carcinoma with > 2% mutation frequency (Fig. 3A). Amplification, deep deletion, splice mutation, and truncating mutation are the primary types of frequent genetic alterations of PTPN3 (Fig. 3B). The mutation sites, types, and sample numbers of the PTPN3 genetic alterations are further presented in Fig. 3C. Specific mutation profiling indicated that out of the total PTPN3 mutations in the database, 79.05% were missense, 20.548% were truncating, and 4.29% cases were splice mutations. There are two primary categories of PTPN3 genetic alterations observed in three distinct cases of Uterine Corpus Endometrial Carcinoma (UCEC): missense mutations and truncating mutations. Specifically, the A716T and R878 alterations were identified in these patients. The most frequently observed putative copy-number modifications of PTPN3 included gain-of-function, amplification, and diploid, as depicted in Fig. 3D. Genomic mutation co-occurrence investigation showed alterations of several genes, including MIR29B2CHG, GVINP1, OR5E1P, KRT8P41, TMEM9B-AS1, OR56A5, and HBBP1, have more commonly detected in the PTPN3-altered group. However, TP53, TTN, and MUC16 were the most frequently mutated genes in PTPN3-altered and non-altered cohorts (Fig. 3E).

### Prognostic significance of PTPN3

**Fig. 4:**
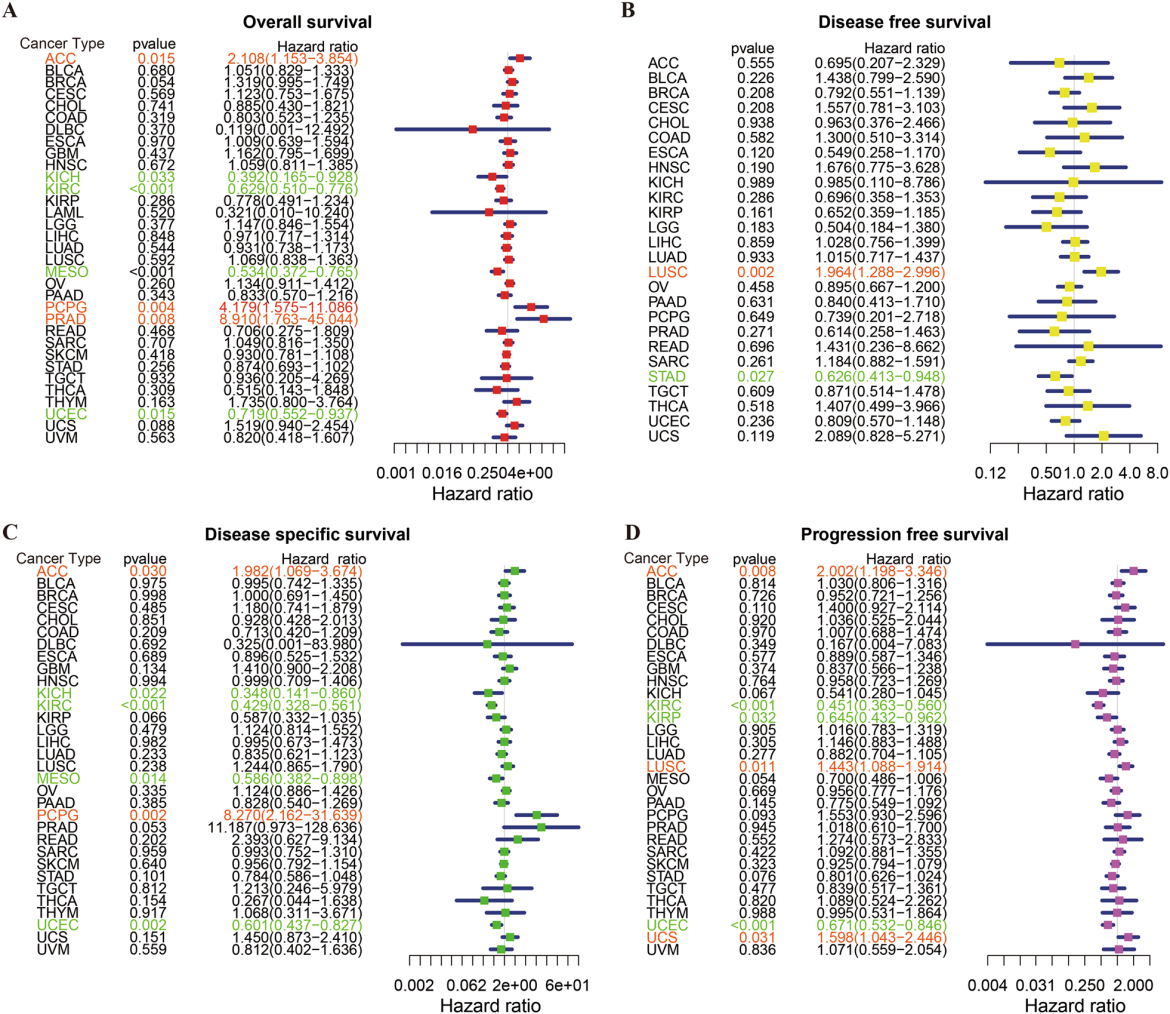
Forest map of univariate Cox regression analysis of PTPN3. **A** Forest map shows the results of univariate Cox regression analysis of PTPN3 for OS in TCGA pan-cancer. **B** Forest map shows the results of univariate cox regression analysis of PTPN3 for DFS in TCGA pan-cancer. **C** Forest map shows the results of univariate Cox regression analysis of PTPN3 for DSS in TCGA pan-cancer. **D** Forest map shows the results of univariate cox regression analysis of PTPN3 for PFS in TGCA pan-cancer. Red items indicate statistical significance

We further explored the prognostic significance of PTPN3 in pan-cancer. The univariate Cox regression analysis showed that PTPN3 was correlated with OS in 7 tumors, DFS in 2 tumors, DSS in 6 tumors, and PFS in 6 tumors (Fig. 4). The findings of univariate Cox regression analysis indicated that the PTPN3 was strongly related to OS in ACC (HR, 2.108; 95% CI 1.153–3.854; *p* = 0.015), KICH (HR, 0.392; 95% CI 0.165–0.928; *p* = 0.033), KIRC(HR, 0.629; 95% CI 0.510–0.776; *p* < 0.001), MESO (HR, 0.534; 95% CI 0.372–0.765; *p* < 0.001), PCPG (HR, 4.179; 95% CI 1.575–11.086; *p* = 0.004), PRAD (HR, 8.910; 95% CI 1.763–45.044; *p* = 0.008) and UCEC (HR, 0.719; 95% CI 0.552–0.937; *p* = 0.015) (Fig. 4A). Furthermore, PTPN3 was highly risky in ACC, PCPG, and PRAD, while it was lowly risky in KICH, KIRC, MESO, and UCEC. The DFS analysis revealed that PTPN3 could act as a high-risk factor in LUSC (HR, 1.964; 95% CI 1.288–2.996; *p* = 0.002), while a low-risk factor in STAD (HR, 0.626; 95% CI 0.413–0.948; *p* = 0.027) (Fig. 4B). The DSS analysis revealed that PTPN3 could act as a risk factor for patients with ACC (HR, 1.982; 95% CI 1.069–3.674; *p* = 0.030), PCPG(HR, 8.270; 95% CI 2.162–31.639; *p* = 0.002), while a protective factor for patients with KICH (HR, 0.348; 95% CI 0.141–0.860; *p* = 0.022), KIRC (HR, 0.429; 95% CI 0.328–0.561; *p* < 0.001), MESO (HR, 0.586; 95% CI 0.382–0.898; *p* = 0.014) and UCEC(HR, 0.601; 95% CI 0.437–0.827; *p* = 0.002) (Fig. 4C). In addition, the PFS analysis suggested that PTPN3 serves a risk role in patients with ACC (HR, 2.002; 95% CI 1.198–3.346; *p* = 0.008), LUSC (HR, 1.443; 95% CI 1.088–1.914; *p* = 0.011) and UCS (HR, 1.598; 95% CI 1.043–2.446; *p* = 0.031), while a protective role in patients with KIRC (HR, 0.451; 95% CI 0.363–0.560; *p* < 0.001), KIRP (HR, 0.645; 95% CI 0.432–0.962; *p* = 0.032) and UCEC (HR, 0.671; 95% CI 0.532–0.846; *p* < 0.001) (Fig. 4D).

**Fig. 5:**
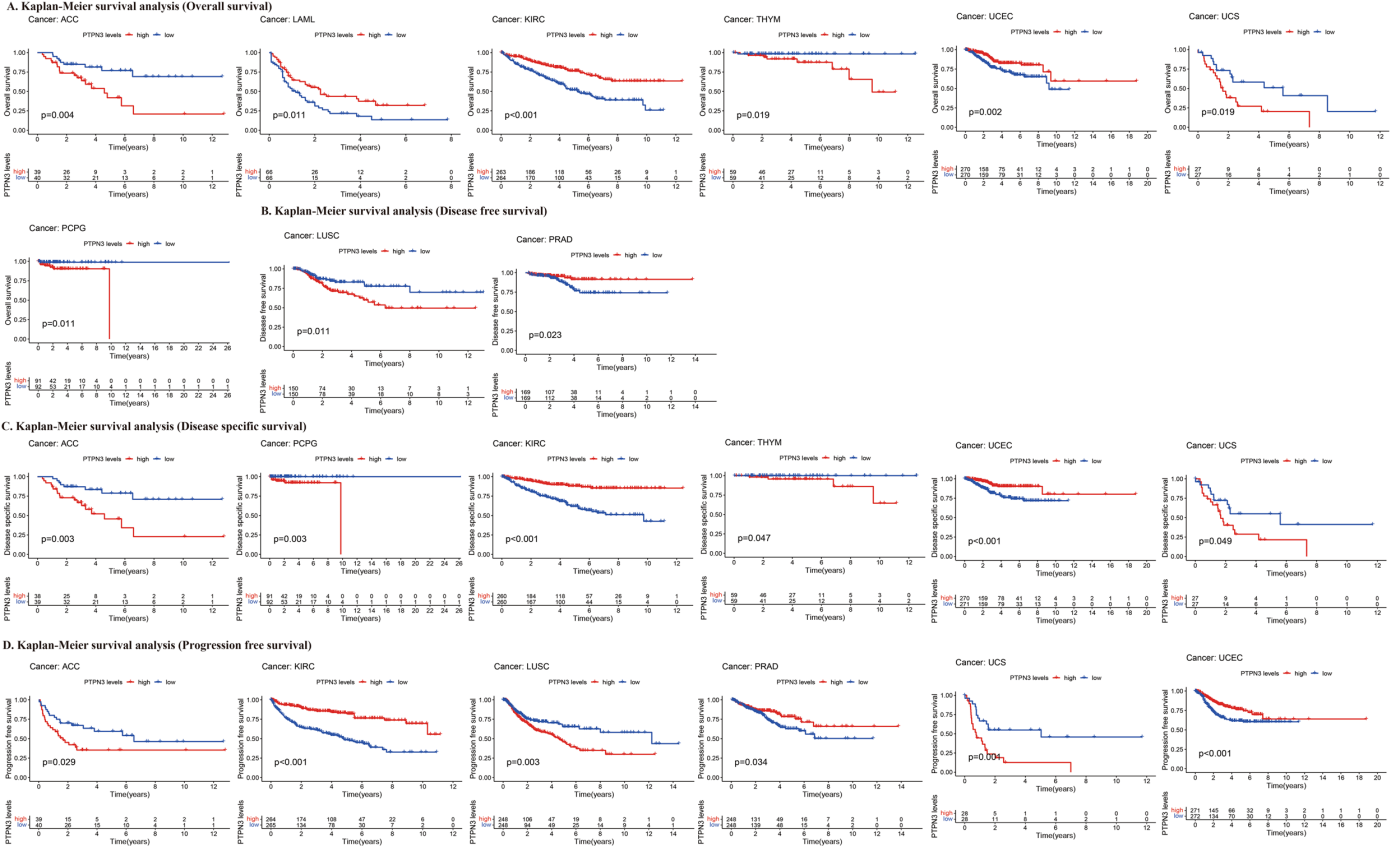
Kaplan–Meier survival curves of PTPN3 in pan-cancer. **A** Kaplan–Meier analysis of the correlation between PTPN3 expression and OS in 7 cancer types. **B** Kaplan–Meier analysis of the correlation between PTPN3 expression and DFS in 2 cancer types. **C** Kaplan–Meier analysis of the correlation between PTPN3 expression and DSS in 6 cancer types. **D** Kaplan–Meier analysis of the correlation between PTPN3 expression and PFS in 6 cancer types

Our results of Kaplan–Meier OS analysis demonstrated that PTPN3 was associated with OS time in 7 cancers, DFS time in 2 cancers, DSS time in 6 cancers, and PFS time in 6 cancers (Fig. 5A–D). A high level of PTPN3 predicted a short survival time in ACC (*p* = 0.004), THYM (*p* = 0.019), UCS (*p* = 0.019), and PCPG (*p* = 0.011), while a high level of PTPN3 predicted long survival time of LAML (*p* = 0.011), KIRC (*p* < 0.001) and UCEC (*p* = 0.002) by Kaplan–Meier survival analysis (Fig. 5A). Our analysis of DFS demonstrated that among patients with LUSC (*p* = 0.011), a poor DFS showed in those with high levels of PTPN3 expression; by contrast, in patients with PRAD (*p* = 0.023), high PTPN3 expression levels were associated with good DFS (Fig. 5B). Besides, we found that higher levels of PTPN3 expression in patients with ACC (*p* = 0.003), PCPG (*p* = 0.003), THYM (*p* = 0.047), and UCS (*p* = 0.049) predicted significantly lower DSS, which was opposite in patients with KIRC (*p* < 0.001) and UCEC (*p* < 0.001) (Fig. 5C). Meanwhile, elevated levels of PTPN3 expression were linked to improved PFS in KIRC (*p* < 0.001), PRAD (*p* = 0.034), and UCEC (*p* < 0.001) but to worse PFS in ACC (*p* = 0.029), LUSC (*p* = 0.003), UCS (*p* = 0.004) (Fig. 5D). Taken together, our findings illustrated the prognostic value of PTPN3 in several kinds of cancers, such as ACC, KIRC, and UCEC.

### Correlation of PTPN3 expression with the tumor immunity

**Fig. 6:**
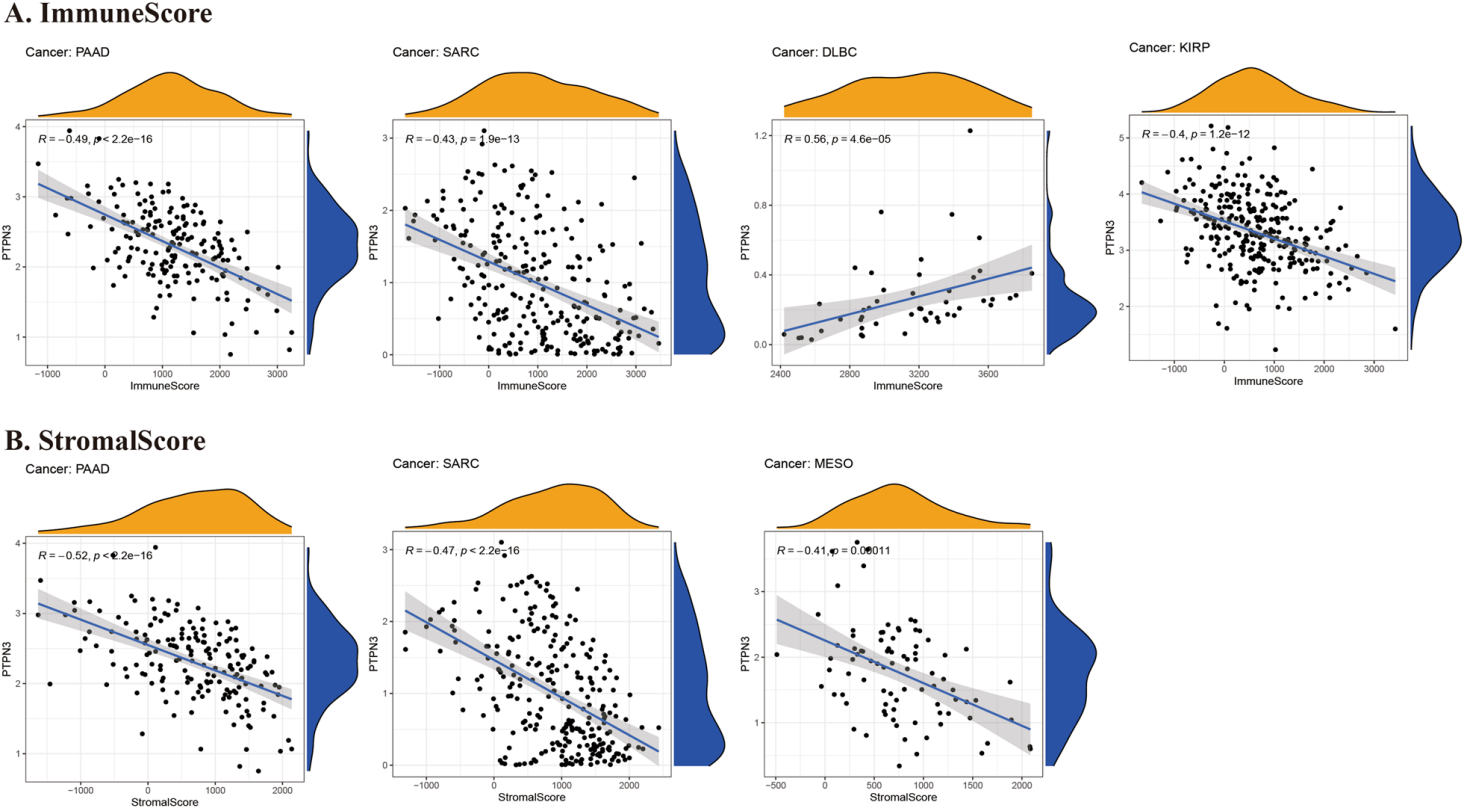
Correlation between PTPN3 and immune and stromal scores in pan-cancer

**Fig. 7:**
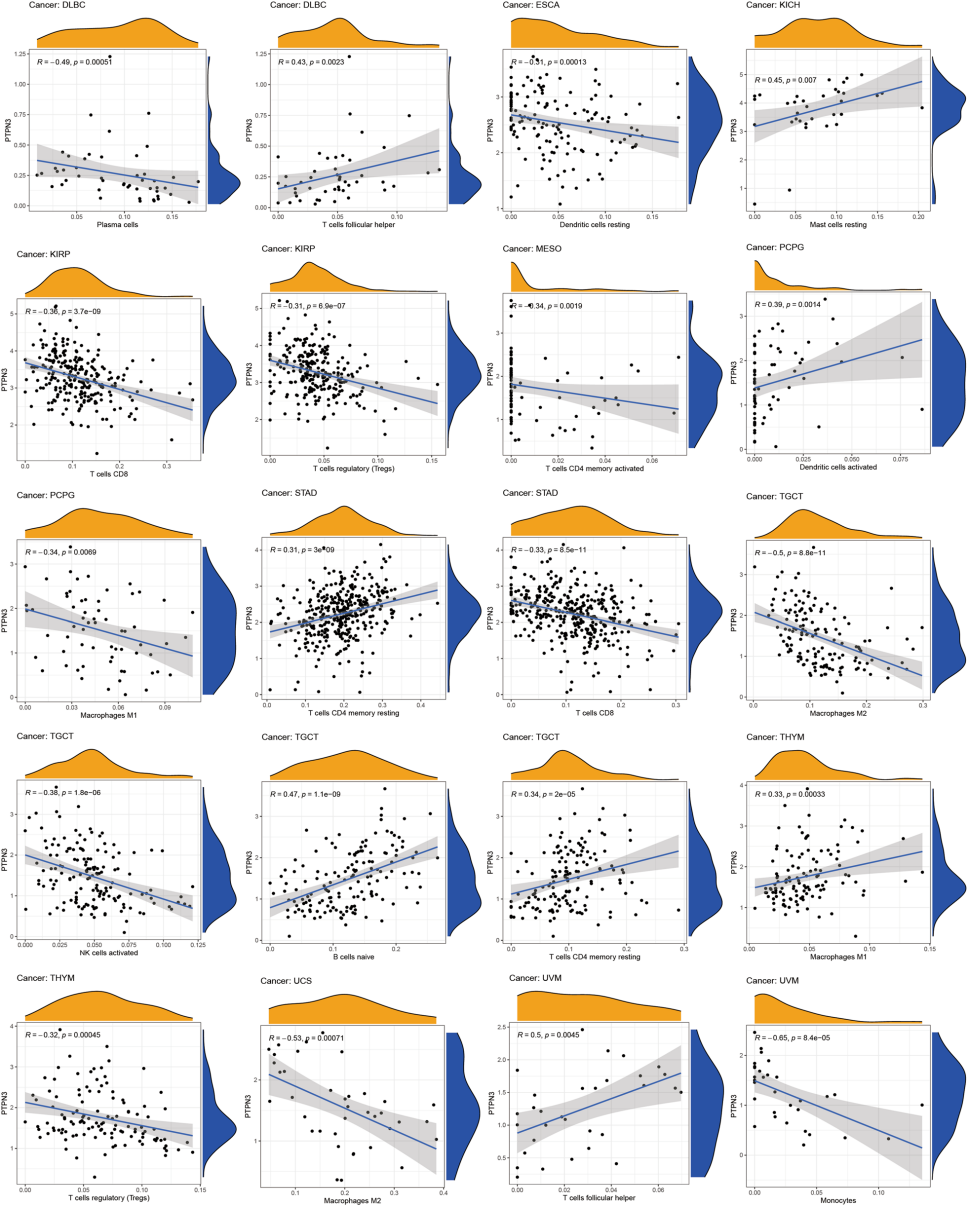
Correlation between PTPN3 expression and the immune cells infiltration in pan-cancer

**Fig. 8:**
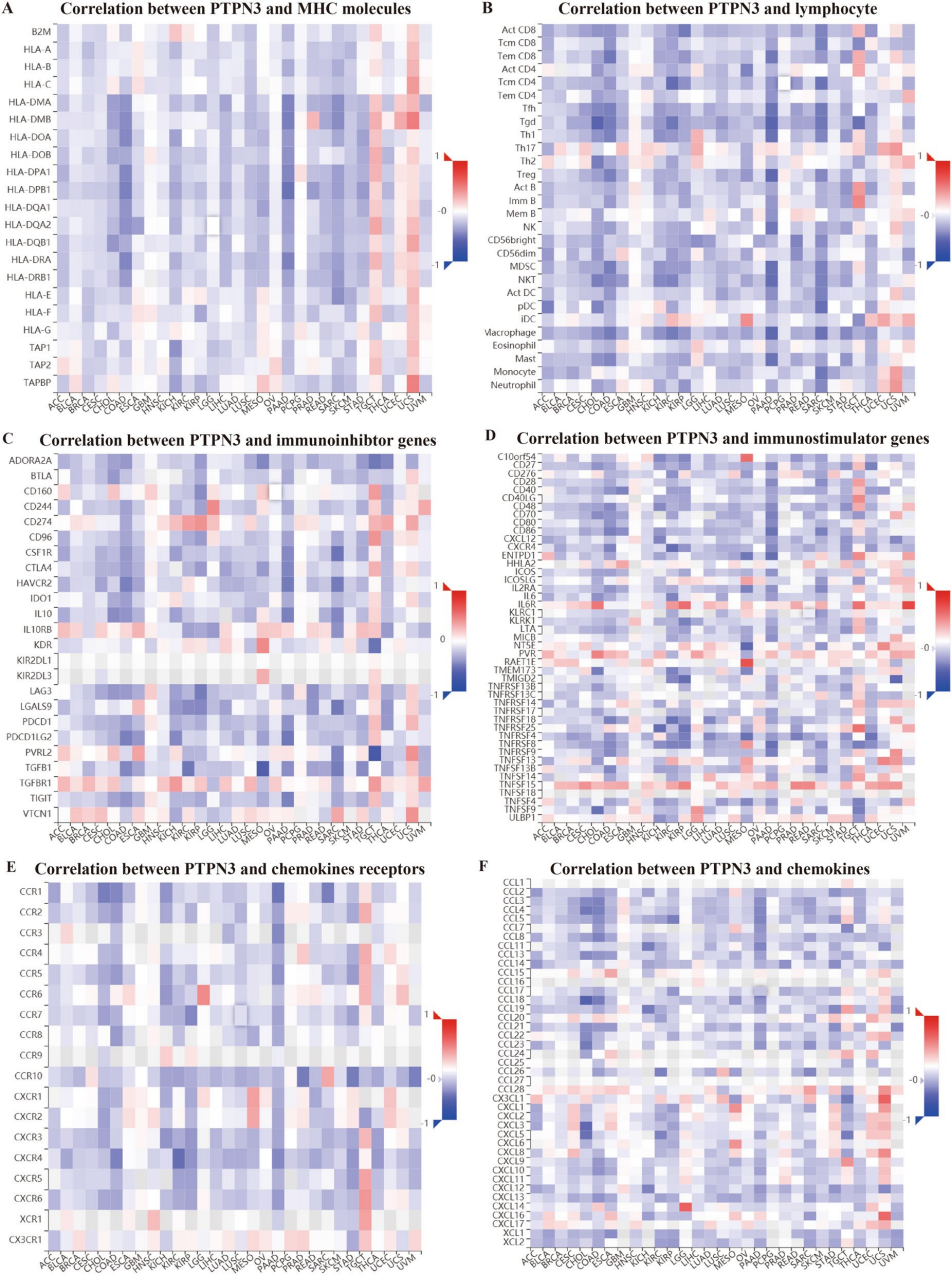
Correlation between PTPN3 expression and immune-related biomarkers in the TISIDB database. The co-expression heatmaps show the association between PTPN3 expression and **A** MHC molecules, **B** lymphocyte, **C** immune-inhibitor genes, **D** immune-stimulator genes, **E** chemokines receptors, and **F** chemokines in pan-cancer

Evidence indicates that the tumor immune microenvironment plays a key role in tumor initiation, progression, and poor prognosis. Within the tumor immune microenvironment, infiltrating immune cell accretion is associated with the tumor immune escaping and cancer progression. Hence, illustrating the relationship between PTPN3 and tumor immune microenvironment is of great importance. We performed the ESTIMATE algorithm to calculate immune scores and explored the associations between immune scores and PTPN3. The findings indicated a negative correlation between PTPN3 and immune scores in PAAD, SARC, and KIRP, while a positive correlation was observed in DLBC (Fig. 6A). In addition, PTPN3 expression and stromal scores were negatively correlated with several cancers, including PAAD, SARC, and MESO (Fig. 6B). Subsequently, we conducted further validation to explore the correlation between the expression of PTPN3 and the levels of infiltration exhibited by 20 immune cells pertinent to the study. The findings demonstrate a substantial correlation between the expression levels of PTPN3 and the infiltration of immune cells across most cancers, as depicted in Fig. 7. We detected that the PTPN3 expression was positively associated with macrophages M1 in THYM, B cells naïve in TGCT, dendritic cells activated in PCPG, memory resting CD4 + T cells in STAD and TGCT, follicular helper T cells in UVM and DLBC, resting mast cells in KICH while negatively associated with macrophages M1 in PCPG, macrophages M2 in TGCT and UCS, regulatory T cells in KIRP and THYM, CD8 + T cells in KIRP and STAD, monocytes in UVM, plasma cells in DLBC, activated NK cells in TGCT, resting dendritic cells in ESCA and memory activated CD4 + T cells in MESO. Next, we investigated the associations between the expression level of PTPN3 and the expression levels of major histocompatibility complex (MHC) genes (Fig. 8A), lymphocyte (Fig. 8B), immuno-inhibitor genes (Fig. 8C), immuno-stimulator genes (Fig. 8D), chemokine receptors (Fig. 8E), and chemokine (Fig. 8F). The findings of our study demonstrate a clear association between the expression of PTPN3 and immune-inhibitor genes, such as PVRL2 and TGFB1, as well as multiple immuno-stimulator genes, such as IL6R, NT5E, RAET1E, TMIGD2, and chemokines including CCL18, CX3CL1, CXCL3, CXCL5, CXCL14, and CXCL16 in various types of cancer. These results suggest the presence of interactions between PTPN3 and immune-related biomolecules in human malignancies.

### Gene set enrichment analysis

**Fig. 9:**
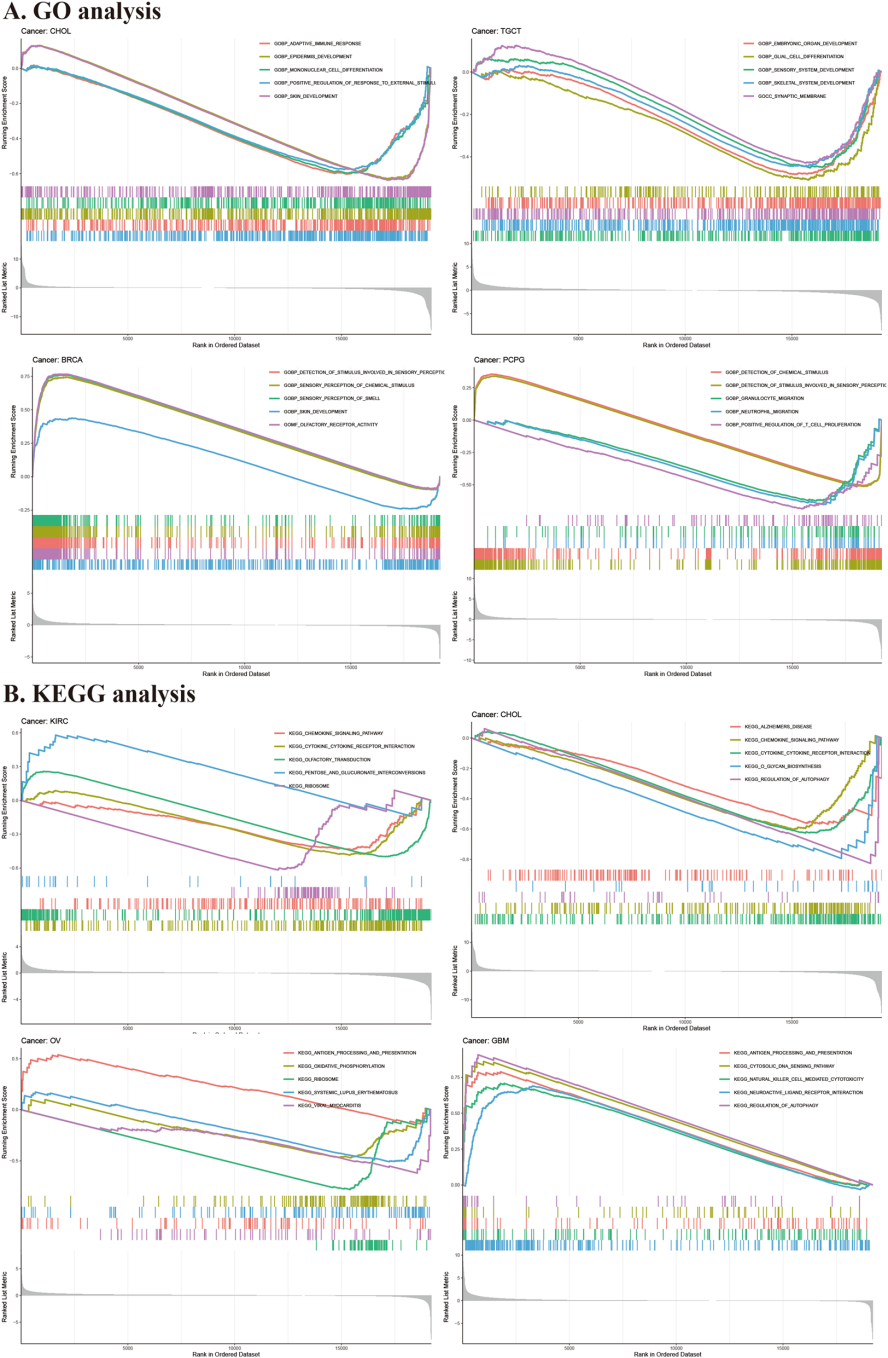
GSEA of PTPN3. **A** GO analysis. **B** KEGG analysis

To identify the primary biological process affected by PTPN3 in pan-cancer, we conducted a biological function analysis of PTPN3 in Cancer GSEA, including GO functional annotation and KEGG pathway analysis (Fig. 9). Notably, GO functional annotation of PTPN3 suggested that PTPN3, across 5 cancers (CHOL, LUSC, MESO, STAD, UVM) are enriched in shared biological processes: adaptive immune response. We also found that PTPN3 was significantly enriched in immune-related biological functions in several cancers, such as granulocyte migration, neutrophil migration, and positive regulation of T cell proliferation in PCPG, activation of the immune response, and B cell activation in THCA, leukocyte cell–cell adhesion and T cell activation in UCS.

KEGG enrichment term exhibited that expression of PTPN3 was mainly associated with 4 pathways, of which the most common signaling pathway was cytokine–cytokine receptor interaction (in BLCA, CHOL, KIRC, LUSC, MESO, STAD, THCA, UVM), followed by antigen processing and presentation (in CESC, LUSC, MESO, DLBC, GBM, OV, UCS). The biological analysis of pan-cancer revealed the involvement of PTPN3 in the regulation of autophagy and drug metabolism cytochrome. Above all, PTPN3 may have a significant role in controlling the tumor immune microenvironment, cytokine–cytokine receptor interaction, and antigen processing and presentation in a variety of malignancies.

### Correlation of PTPN3 with TMB and MSI

**Fig. 10:**
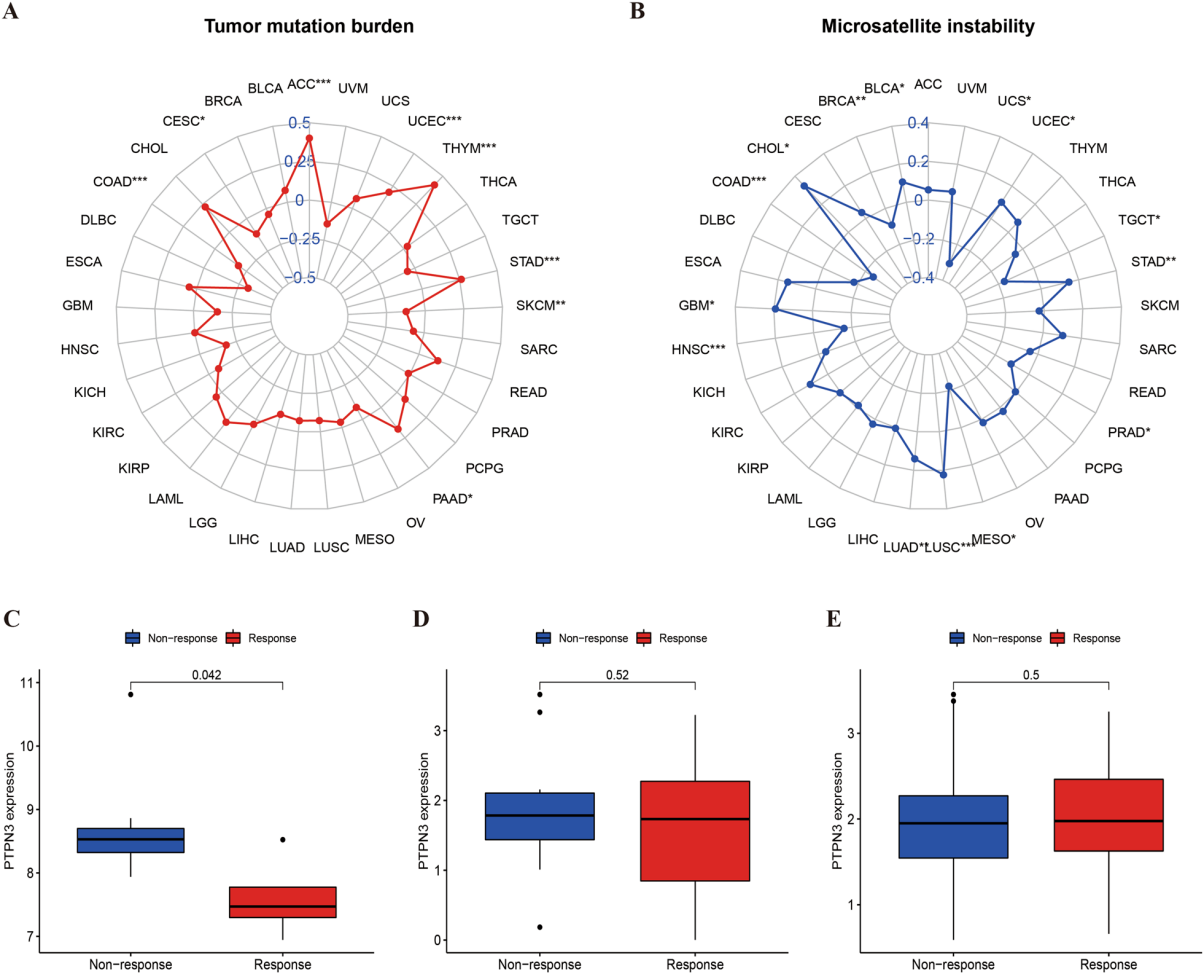
Correlation between PTPN3 expression and TMB levels, MSI event, and immunotherapeutic efficacy. **A** Radar map of the relationship between PTPN3 expression and TMB levels. **B** Radar map of the relationship between PTPN3 expression and MSI event. **C**–**E** Relationship between PTPN3 expression and the immunotherapeutic efficacy in GSE67501 **C**, GSE78220 **D**, and Imvigor **E**

TMB and MSI are two emerging biomarkers associated with the immunotherapy response of cancer patients. Thus, we studied the correlation between PTPN3 expression and TMB/MSI across several tumors of TCGA (Fig. 10). We found an association between PTPN3 expression and TMB in 8 cancers, suggesting that the overexpression of PTPN3 was positively connected with TMB in ACC, UCEC, THYM, STAD, and PAAD while negatively correlated with TMB in COAD, CESC, and SKCM (Fig. 10A). In addition, we also detected an association between PTPN3 expression and MSI in 14 cancers. The result displayed that PTPN3 was positively correlated with upregulated MSI in GBM, CHOL, BLCA, UCEC, STAD, LUSC, and LUAD while negatively associated with MSI in COAD, BRCA, UCS, TGCT, PRAD, MESO and HNSC (Fig. 10B). These results indicated that PTPN3 may significantly correlate with patients' responses to immunotherapies.

### Correlation of PTPN3 with the immunotherapeutic efficacy

To explore the promising utility of PTPN3 in predicting response to immunotherapeutic in some tumors, GEO data sets, including GSE67501, GSE78220, and Imvigor, were collected for analysis. Figure 10C, D and E illustrates the differences in PTPN3 expression between patients who responded to immunotherapy and those who did not respond to immunotherapy. The results of our study revealed a statistically significant increase in PTPN3 expression in the non-response group of the GSE67501 cohort (*p* = 0.042) (Fig. 10C). This discovery suggests that PTPN3 may serve as a prognostic marker for the response to immunotherapy and has potential therapeutic application.

### Correlation of PTPN3 with drug sensitivity

**Fig. 11:**
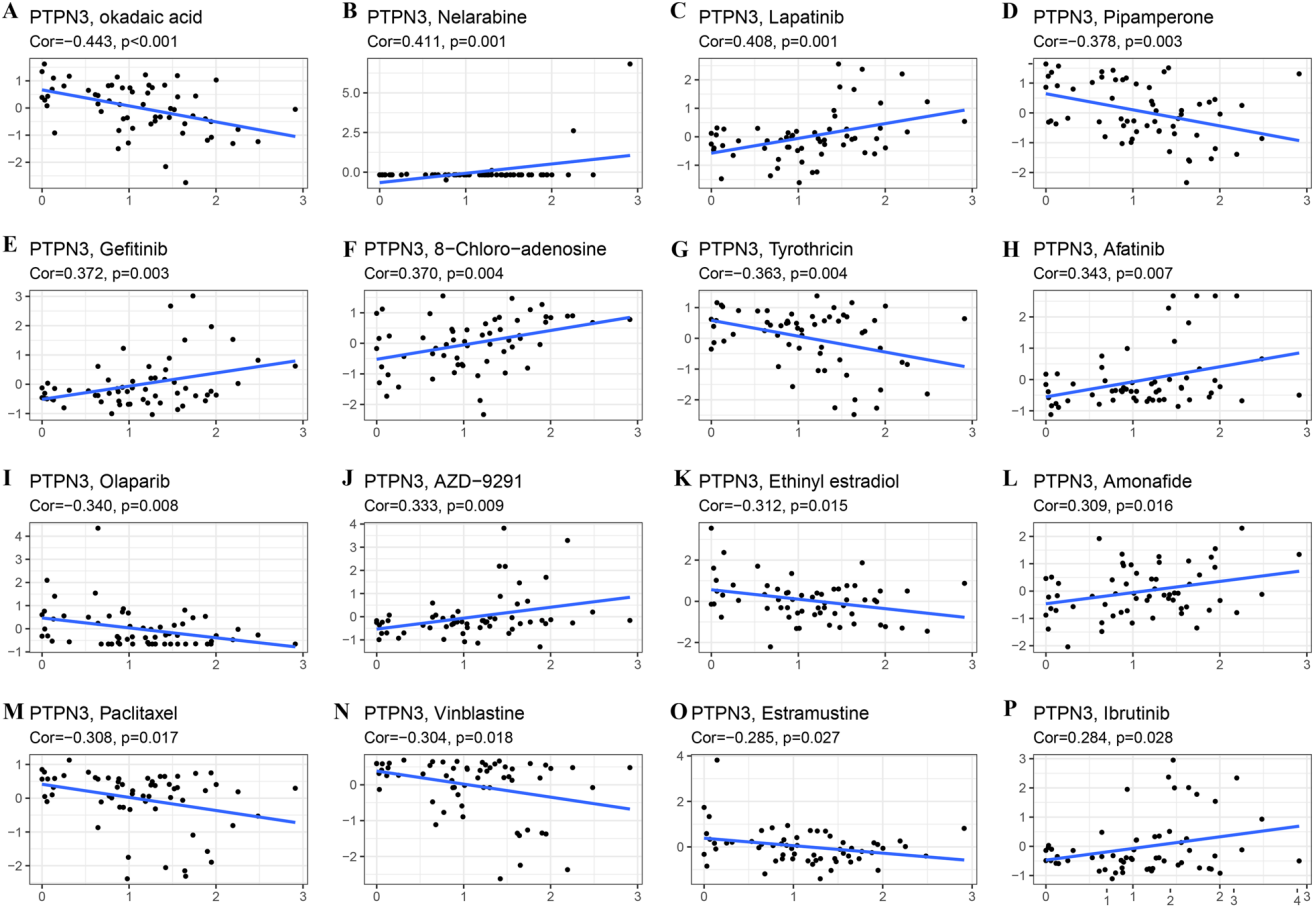
Correlation between PTPN3 expression and drug sensitivity. The PTPN3 was linked to the sensitivity of **A** Okadaic acid, **B** Nelarabine, **C** Lapatinib, **D** Pipamperone, **E** Gefitinib, **F** 8-Chloro-adenosine, **G** Tyrothricin, **H** Afatinib, **I** Olaparib, **J** AZD-9291, **K** Ethinyl estradiol, **L** Amonafide, **M** Paclitaxel, **N** Vinblastine, **O** Estramustine, **P** Ibrutinib

We further investigated the correlation between PTPN3 expression and drug sensitivity in CellMiner™ online database. PTPN3 expression was negatively related to the sensitivity to okadaic acid (Fig. 11A), pipamperone (Fig. 11D), tyrothricin (Fig. 11G), olaparib (Fig. 11I), ethinyl estradiol (Fig. 11K), paclitaxel (Fig. 11M), vinblastine (Fig. 11N) and estramustine (Fig. 11O). In contrast, PTPN3 expression was positively associated with the sensitivity to nelarabine (Fig. 11B), lapatinib (Fig. 11C), gefitinib (Fig. 11E), 8-Chloro-adenosine (Fig. 11F), afatinib (Fig. 11H), AZD-9291 (Fig. 11J), amonafide (Fig. 11L) and ibrutinib (Fig. 11P). The findings of the study revealed a significant correlation between PTPN3 and the responsiveness of various chemotherapeutic drugs frequently employed in clinical settings, including paclitaxel, lapatinib, and vinblastine.

### IHC validation of PTPN3

**Fig. 12:**
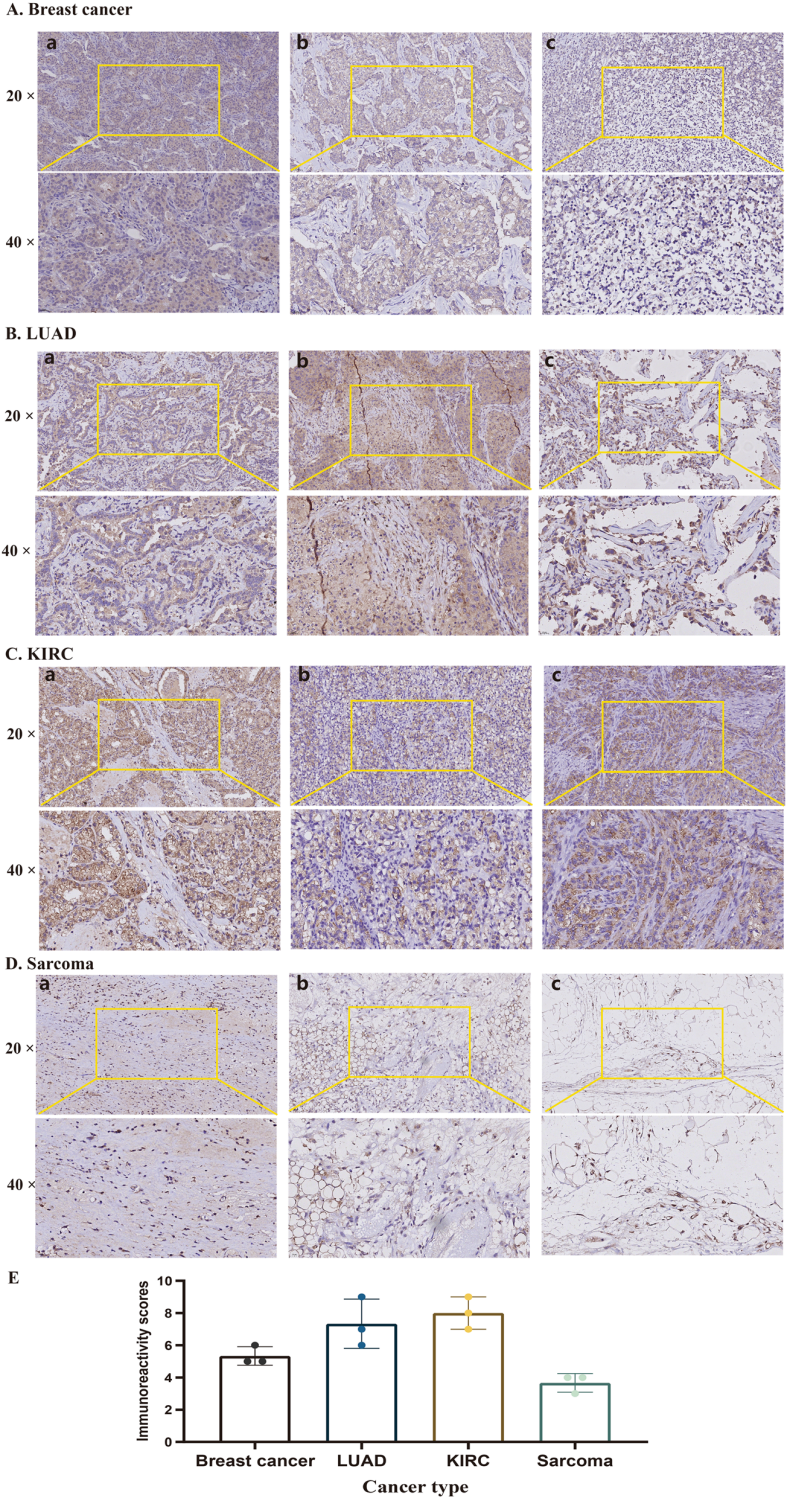
Immunohistochemistry validation of PTPN3 in pan-cancer by clinical samples. **A** Breast cancer. **B** LUAD. **C** KIRC. **D** Sarcoma. **E** Immunohistochemistry scores of each cancer

We further validated PTPN3 expression by IHC in 4 different cancers by our cohorts, including breast cancer, lung adenocarcinoma, kidney renal clear cell carcinoma, and sarcoma. PTPN3 was positively detected in cancer samples. A strongly positive expression of PTPN3 was observed in KIRC, and LUAD, followed by a moderately positive expression of PTPN3 in breast cancer. Weakly positive PTPN3 expression was detected in patients with sarcoma (Fig. 12A–D). The immunohistochemistry scores of PTPN3 in different cancers are presented in Fig. 12E. The IHC results were consistent with previous findings by bioinformatics analysis and further validated the expression levels of PTPN3 in human malignancies.

## Discussion

PTPN3 is a member of the non-transmembrane protein phosphatase (PTP) family and plays a crucial role in the MAPK and PI3K signaling pathways in MAPK and PI3K signaling pathways through tyrosine kinase phosphorylation. According to numerous lines of evidence, PTPN3 is abnormally elevated in several malignancies, and can promote cancer progression, metastasis, and therapy resistance. It has been found that PTPN3 inhibition significantly contributes to enhancing the activation and functions of activated lymphocytes, and has anti-cancer effects on small-cell lung cancer (SCLC) and large-cell neuroendocrine carcinoma (LCNEC) [21, 22]. However, findings from several studies have indicated that PTPN3 can serve as a tumor suppressor during cancer development. We carried out a thorough investigation in the current pan-cancer research to highlight the crucial functions that PTPN3 plays in immunological activity, immunotherapy, and cancer prognosis.

First, we explored the expression pattern and clinical value of PTPN3 in human malignancies. Compared to the corresponding adjacent normal tissues of the TCGA pan-cancer data set, we found a higher PTPN3 expression in BLCA, CESC, KICH, LUSC, STAD, and UCEC, and a lower PTPN3 expression in BRCA, GBM, HNSC, KIRC, KIRP and LIHC. Through a comparison of PTPN3 expression in tumor samples from The Cancer Genome Atlas (TCGA) and normal samples from the Genotype-Tissue Expression (GTEx) database, it was shown that COAD, ESCA, LUAD, OV, PAAD, PRAD, TGCT, and UCS exhibited a greater level of PTPN3 expression. Conversely, a lower expression level of PTPN3 was observed in LGG, SKCM, and THCA. Previous research has demonstrated a high expression level of PTPN3 in ESCA, which supports our current findings [23]. Peng et al. also found that PTPN3 was downregulated in KIRC, and restoration of PTPN3 could inhibit tumor cell motility by suppressing the phosphorylation of AKT, subsequently inactivating the PI3K/AKT signaling pathway of renal cell carcinoma cells [24]. Previous studies have found a statistically significant correlation between PTPN3 and the differentiation status of gastric cancer tissue [25]. It has been found that miR-574-5p can inhibit the expression of PTPN3, and promote angiogenesis in gastric cancer cells by enhancing phosphorylation of p44/42 MAPK [26]. However, Wang et al. found that the expression of PTPN3 in GBM tissues was significantly higher and served as an independent prognostic factor in GBM [27], which differed from our results. In addition, we found that the differential PTPN3 expression between BRCA samples and normal samples obtained from the TGCA or GTEx database showed opposite results. A previous study has indicated that PTPN3 was overexpressed in breast cancer, and stimulates breast cancer growth through regulating vitamin D receptor (VDR) expression [28]. Moreover, our findings indicate that PTPN3 was upregulated in later clinical stages in ACC and COAD, while it was significantly downregulated in later stages in KIRC and TGCT, suggesting that monitoring PTPN3 expression may predict cancer progression in clinical settings. Some inconsistencies mentioned above may be due to the following reasons: different algorithms, sample sizes, and sources in different databases, and currently, there is a lack of criteria for objectively evaluating the quality of a single database; the insufficient number of normal samples in TCGA may also lead to the detection of some cancer types not being significant; and there may be batch effects which can be attributed to a different time, data set, laboratory, platform, and other factors; in addition, gene expression may alter during cancer development and after receiving various therapies; it is worth noting that genes do not function alone, and may be passively regulated in tumorigenesis or tumor progression. Therefore, future studies are warranted to validate the expression pattern of PTPN3 in these cancer types and should be further validated in larger cohorts with identity and modeled to remove batch effects and optimize standardized algorithms.

Cancer patients have genomes that contain an average of 4–5 driver mutations when combining coding and non-coding genomic regions, indicating that genetic changes play a key role in cancer initiation and development [29]. The genetic alteration characteristics of PTPN3 among malignancies, however, remain unknown. Our study found that the total alteration rate of the PTPN3 gene was nearly 2.2% in pan-cancer, and a variety of non-synonymous mutations of PTPN3, including missense, amplification, deep deletion, splice mutation, and truncating mutation, have been detected in different cancers. A comprehensive investigation of the tyrosine phosphatase gene superfamily in pan-cancer has confirmed the existence of 83 somatic mutations in diverse PTP family members; these mutations have been found to affect roughly 26% of colorectal malignancies [30]. From whole exome sequencing analysis, Gao et al. found that more than 40% of intrahepatic cholangiocarcinoma samples contain somatic mutations, and a frequent mutation of PTPN3–L232R was demonstrated to induce cholangiocarcinoma cell proliferation and migration and increased risk of tumor recurrence in patients [12]. The potential roles of PTPN3 mutations in human cancer are warranted for in-depth investigation in future studies.

Concerning the prognostic significance of PTPN3 in pan-cancer, our findings indicated that PTPN3 is a protective prognostic factor for patients with LAML, KIRC, and UCEC and a risk factor for patients with ACC, THYM, UCS, and PCPG in OS analysis. Regarding DFS, our results revealed that patients with high PTPN3 expression had a longer survival time in PRAD and a shorter survival time in LUSC. As for PFS analysis, PTPN3 can act as a protective factor in KIRC, PRAD, and UCEC and a risk factor in ACC, LUSC, and UCS. Consistent with our findings, prior research has indicated that PTPN3 overexpression could inhibit kidney cancer progression by suppressing the AKT signaling pathway and served as a favorable prognostic factor in patients with KIRC [24], which is consistent with our results. We also found that PTPN3 is a risk factor in LUSC in both DFS and PFS analysis. Previous research has indicated that PTPN3 plays a pivotal role as an immune checkpoint in activated lymphocytes, and inhibition of PTPN3 in lymphocytes expands the proportion of tumor-infiltrating lymphocytes and activated lymphocyte cytotoxicity, thus exerting the anti-cancer effects on SCLC and LCNEC progression [21, 31]. In this way, the body's immune response may have a significant influence on the relationship between PTPN3 expression and cancer prognosis. Put together, PTPN3 may play diverse functions in pan-cancer and is a significant prognostic biomarker in multiple human cancers; evaluating PTPN3 expression may aid in predicting the prognosis of cancer patients, as supported by prior research.

Next, we investigated PTPN3's potential downstream pathways and potential roles in biological processes. Our GO analysis findings suggested that PTPN3 is enriched in immune-modulatory functions, including adaptive immune response, immune cell migration and regulation, and activation of the immune response. The KEGG analysis revealed that PTPN3 was enriched in the cytokine–cytokine receptor interaction pathway, and the antigen processing and presentation pathway. PTPN3 has been extensively studied about T-cell signal transduction and lymphocyte activation. According to previous reports, PTPN3 is essential for immune checkpoint function in activated lymphocytes, where transcription factor NF-κB enhances PTPN3 expression. On the other hand, TGF-β decreases PTPN3 expression and NF-κB activation in the cancer microenvironment and prevents lymphocytes from functioning biologically [21, 32]. Several investigations have demonstrated that PTPN3 is involved in reducing T cell activation and adversely modulating TCR signaling [33, 34]. These findings are consistent with our findings and support the crucial roles of PTPN3 in tumor immunity. However, the fundamental mechanisms by which PTPN3 regulates tumor immunity during cancer progression are unknown and need to be validated further through research.

Tumor immune cell infiltration is an important signal of host immunological responses to cancer cells and is associated with cancer development and prognosis [35]. Exploring immune cell infiltration-related indicators may, therefore, give novel strategies for regulating tumor immunity and improving treatment efficacy. Our findings reveal that PTPN3 is significantly connected to the quantity of macrophages, T cells, and B cells in diverse cancer types. According to our findings, there is a link between PTPN3 expression and the invasion of naive B cells and memory-resting CD4 + T cells in TGCT. In contrast, we discovered a negative relationship between PTPN3 expression and the infiltration of M2 macrophages and activated NK cells in TGCT. PTPN3 expression was associated with memory-resting CD4 + T cells in STAD. Evidence has shown the significant prognostic value of the abundance of CD4 + [36] and CD8 + T cells [37, 38] in cancer patients. For instance, STAD patients with high CD8 + T cell infiltration had significantly better prognosis [38]. Likewise, well-differentiated STAD patients have higher levels of tumor‐infiltrating CD4 + T cells [39]. These findings further confirm the potential roles of PTPN3 in mediating T-cell functions in tumor immune microenvironment, thereby affecting cancer progression and prognosis. Combined with the significant involvement of PTPN3 in immune-related biological functions and signaling pathways by GO and KEGG analyses mentioned above, we imply that PTPN3 acts as an essential regulator of tumor immunity may influence the prognosis and therapy responses of cancer patients through mediating immune microenvironment, which requires a further understanding of the immunological activity of PTPN3.

TMB and MSI are essential indicators of sensitivity to immunotherapies [40, 41]. Among 33 cancer types, we found that PTPN3 is connected with TMB in 8 cancer types and MSI in 14 cancer types, indicating the promising potential of PTPN3 as a predictive biomarker in immunotherapy efficacy. Previous research has revealed the genetic alterations of PTP genes in MSI-High cancer cell lines [42]. Moreover, mounting data suggest that the PTPN family may have a major role in the effectiveness of immunotherapy, including CAR-T treatments and immune checkpoint inhibitors. PTPN11 has been reported to participate in tumor immunotherapy resistance and has spawned several inhibitors, which can be applied in combination with anti-PD-1 and anti-PD-L1 therapies to improve immunotherapy efficacy [43–45]. Although PTPN3 is closely related to the activation and infiltration of immune cells, its relationship with tumor immunotherapy is still largely unknown, with a great development prospect. Our result revealed that PTPN3 expression was significantly higher in renal cell carcinoma patients who did not respond to anti-PD-1/PD-L1 treatment. Nowadays, the advent of immunotherapy based on anti-PD-1/PDL-1 agents has provided a survival advantage in patients with kidney cancer [46]. Based on these findings, PTPN3 may be related to the PD-1/PD-L1 axis, which may facilitate predicting immunotherapy efficacy with promising clinical applications; however, it still needs further validation.

Moreover, several studies have investigated the potential roles of the PTPN family in mediating cancer drug resistance. PTPN3 expression was dramatically elevated in both cisplatin- and doxorubicin-resistant ovarian cancer cells compared to parental cells, and inhibiting PTPN3 restored sensitivity to both drugs [47]. Furthermore, PTPN3 has been reported to dephosphorylate EGFR, thus increasing sensitivity to tamoxifen and tyrosine kinase inhibitors (TKIs) in breast cancer [48, 49]. Our findings reveal that PTPN3 can diminish the sensitivity of certain anti-cancer medications, including paclitaxel, okadaic acid, pipamperone, and tyrothricin while increasing the sensitivity of others, including nelarabine, afatinib, lapatinib, and gefitinib. Afatinib is an orally effective epidermal growth factor receptor (EGFR) tyrosine kinase inhibitor (TKI) used in the treatment of ERBB1-mutant lung cancer, and lapatinib has been widely applied in treating ErbB2-overexpressing breast cancer. Gefitinib has been approved by the FDA for the treatment of advanced NSCLC after other therapeutic approaches have failed. Hence, PTPN3 may play important roles in mediating targeted therapy resistance in human cancers, and monitoring PTPN3 expression may facilitate choosing the most effective therapy strategy for individual patients. Our findings provide new evidence for the associations of PTPN3 with anti-cancer drug sensitivity, and targeting PTPN3 may be a practical approach to reverse cancer drug resistance.

Despite completing extensive research to investigate and analyze the biological activities of PTPN3 in cancer progression, our current study is primarily based on bioinformatics and has certain limitations. First, while the expression pattern in our clinical samples has been validated, the predictive significance of PTPN3 and its associations with immunotherapeutic efficacy and anti-cancer medicine sensitivity in our cohorts has yet to be proven. Besides, the specific process by which PTPN3 regulates the tumor immune microenvironment remains largely unknown, and the potential role of PTPN3 in modulating tumor immunity has not been investigated through in vivo and in vitro research. Hence, more in-depth studies are needed to clarify the mechanism and biological roles of PTPN3 in tumor immunity. In addition, combining the molecular investigation of PTPN3 in malignancies and macroscopic imaging studies may facilitate the early diagnosis and the personalized treatment approach, which can be further investigated in future studies [50].

## Conclusions

This study provided evidence for the prognostic value of PTPN3 in pan-cancer, and its critical roles in mediating cancer drug sensitivity, tumor immune microenvironment, and immunotherapy efficacy, providing a novel biomarker and immunotherapeutic target for cancer treatment and facilitating the development of more precise and personalized immunotherapy strategies.

## Data Availability

The original contributions presented in the study are included in the article/Supplementary Material, further inquiries can be directed to the corresponding authors.

## Abbreviations

PTPN3: Phosphatase non-receptor type 3
TCGA: The Cancer Genome Atlas
IHC: Immunohistochemistry
TMB: Tumor mutation burden
MSI: Microsatellite instability
GSEA: Gene set enrichment analysis
TME: Tumor microenvironment
OS: Overall survival
PFS: Progression-free survival
DFS: Disease-free survival
DSS: Disease-specific survival
HR: Hazard ratio
KEGG: Kyoto encyclopedia of genes and genomes
GO: Gene ontology (GO)
ESTIMATE: Estimation of stromal and immune cells in malignant tumors using expression
SCLC: Small-cell lung cancer
LCNEC: Large-cell neuroendocrine carcinoma
